# Clinical, Biochemical, and Kidney Abnormalities in McKittrick–Wheelock Syndrome: An Individual-Patient Systematic Review of Published Cases

**DOI:** 10.3390/jcm15218374

**Published:** 2026-10-27

**Authors:** Matilde Lepori, Virginia Turner, Massimiliano Bertacchi, Mario G. Bianchetti, Pietro Camozzi, Sebastiano A. G. Lava, Martin Scoglio, Gregorio P. Milani, Johannes M. A. Toti, Lisa Ponti

**Affiliations:** 1 Family Medicine Institute, Faculty of Biomedical Sciences, Università della Svizzera Italiana, 6900 Lugano, Switzerlandmartin.scoglio91@gmail.com (M.S.);; 2 Division of Pediatric Nephrology, Department of Pediatrics, Gynaecology and Obstetrics, Geneva University Hospitals, 1205 Geneva, Switzerland; 3 Department of Anesthesia, Hôpital du Valais, 1951 Sion, Switzerland; 4 Pediatric Cardiology Unit, Centre Hospitalier Universitaire Vaudois, Lausanne University Hospital, and University of Lausanne, 1011 Lausanne, Switzerland; webmaster@sebastianolava.ch; 5 Department of Pharmacy, Health and Nutritional Sciences, University of Calabria, 87036 Rende, Italy; 6 Pediatric Unit, Annunziata Hospital, 87100 Cosenza, Italy; 7 Department of Surgery, Ospedale Regionale di Bellinzona e Valli, EOC, 6500 Bellinzona, Switzerland

**Keywords:** acute kidney injury, colorectal adenoma, diarrhea, electrolyte disorders

## Abstract

**Background/Objectives**: McKittrick–Wheelock syndrome is a rare entity secondary to colorectal tumors, characterized by chronic diarrhea, electrolyte and acid–base disturbances, and impaired kidney function; its pathophysiology and biochemical spectrum remain incompletely defined. **Methods**: We conducted an individual-patient systematic review of published cases (PROSPERO CRD420251233022). Eligible patients had a colorectal tumor, chronic diarrhea, and at least one otherwise unexplained electrolyte or acid–base disturbance. Data on demographics, presentation, tumor characteristics, electrolyte and acid–base abnormalities, creatinine levels, and mechanisms of diarrhea were extracted and analyzed. **Results**: Among 195 reports including 231 patients, median age was 68 years and symptoms usually persisted >12 months. Tumors were predominantly large distal colorectal adenomas: 97% were within 29 cm and 68% within 10 cm of the anal verge; none measured ≤50 mm and 78% measured ≥100 mm. Hypokalemia occurred in 90%, hyponatremia in 91%, metabolic acidosis in 50%, metabolic alkalosis in 28%, and elevated creatinine in 91%. Stool osmotic-gap data in eight patients and reduced stool losses with non-steroidal anti-inflammatory drugs in seven cases were compatible with secretory, partly prostaglandin-mediated diarrhea. No statistically significant, biochemical differences were detected by tumor size. **Conclusions**: McKittrick–Wheelock syndrome is a recognizable, potentially treatable syndrome associated with chronic diarrhea and major metabolic and kidney abnormalities, requiring early recognition.

## 1. Introduction

Colorectal tumors may occasionally present with diarrhea, which in turn can lead to volume depletion, electrolyte and acid–base derangements, and impaired kidney function [1,2,3]. The condition was first described in the 1950s at Massachusetts General Hospital by Leland S. McKittrick (1893–1978) and Frank C. Wheelock Jr. (1919–2006), after whom it was subsequently named [4,5].

The mechanisms linking the underlying tumor to the cascade of fluid losses, electrolyte and acid–base derangements, and kidney dysfunction observed in McKittrick–Wheelock syndrome remain incompletely characterized. A systematic review published in 2018 provided a predominantly qualitative assessment of this disease [5]. The present individual-patient systematic review aims to quantify acid–base, electrolyte, and kidney function abnormalities and explore the mechanisms underlying diarrhea.

## 2. Materials and Methods

### 2.1. Study Preregistration and Data Sources

This patient-level systematic review was registered in the International Prospective Register of Systematic Reviews (PROSPERO; CRD420251233022) and carried out in accordance with the Preferred Reporting Items for Systematic Reviews and Meta-Analyses (PRISMA) 2020 guidelines (Table S1) [6,7]. We searched the U.S. National Library of Medicine (MEDLINE), Excerpta Medica (EMBASE), and Web of Science from inception, without language restrictions. The search strategy combined the terms “McKittrick–Wheelock syndrome”, “McKittrick syndrome”, “Wheelock syndrome”, and “metabolic acid–base disturbance” with “colorectal tumor”. Reference lists of retrieved reports and relevant reviews were also screened to identify additional cases. Eligible publications included original reports and letters to the editor reporting individual cases. Searches were conducted in February 2026 and updated prior to submission (1 July 2026). Titles and abstracts were screened for relevance, followed by full-text assessment of potentially eligible articles.

### 2.2. Selection Criteria and Definitions

McKittrick–Wheelock syndrome was defined in individuals fulfilling all three of the following criteria [2]: presence of a colorectal tumor; tumor-attributable diarrhea persisting for at least 4 weeks; and at least one otherwise unexplained electrolyte disturbance or metabolic acid–base disturbance [8,9,10,11,12].

Patients were excluded when underlying conditions or pharmacological treatments known to cause electrolyte or acid–base derangements were identified. For each case, data were extracted on five domains: (1) demographics and history, with emphasis on symptom duration; (2) symptoms and signs, including abdominal pain, development of symptoms triggered by standing and relieved by recumbency (orthostatic intolerance), fatigue, nausea or vomiting, protrusion of the tumor beyond the anal verge (tumor prolapse), self-reported passage of red blood per rectum (hematochezia), and self-reported unintentional weight loss; (3) tumor location (distance from the anal verge, categorized as ≤5, 6–10, 11–15, 16–29, and ≥30 cm) and size (maximum reported tumor diameter, categorized as ≤50, 51–99, 100–149, 150–199, and ≥200 mm); (4) histopathologic tumor classification (villous adenoma, tubulovillous adenoma, tubular adenoma, adenoma with intramucosal carcinoma, or adenocarcinoma); and (5) laboratory parameters in blood, with emphasis on sodium, potassium, acid–base balance, creatinine, and urea.

The severity and classification of laboratory abnormalities are defined in Table 1.

Because the available data did not permit formal classification of kidney dysfunction as acute or chronic, hypercreatininemia was descriptively classified into predefined categories starting at ≥101 µmol/L (Table 1). These thresholds do not establish a diagnosis of acute kidney injury or chronic kidney disease and should not be interpreted as validated grades. Finally, although rarely available, data on stool water sodium and potassium concentrations were sought to distinguish osmotic from secretory diarrhea, along with information on the symptomatic treatment of diarrhea with nonsteroidal anti-inflammatory drugs.

### 2.3. Completeness of Reporting

Reporting completeness for the five predefined datasets was assessed for each case using a score from 0 to 2 per item. Total scores were categorized as excellent (9–10), good (7–8), or acceptable (5–6). Cases with a score ≤4 were excluded. This score measures reporting completeness rather than study-level risk of bias. No formal risk-of-bias tool was applied to the source case reports.

### 2.4. Data Extraction and Statistical Analysis

Reports were independently screened, data extracted, and reporting completeness assessed in duplicate and without blinding by two investigators, with disagreements resolved by consensus or third-reviewer adjudication when required. Extracted data were recorded in a standardized dataset and verified for consistency. Potential duplicate cases were identified based on explicit statements in the source articles, comparison of demographic and clinical or laboratory characteristics, and cross-checking of referenced publications [13].

Patients were categorized a priori according to tumor size (51–149 mm versus ≥150 mm). To assess potential temporal differences, we used 2000 as a pragmatic cutoff and compared demographic characteristics, laboratory findings, and tumor size between cases published in 1955–1999 and those published in 2000–2026.

Missing data were handled using pairwise deletion [14]. Categorical variables are presented as frequencies and percentages, calculated using the number of cases with available data as the denominator, and were compared using Fisher’s exact test. Continuous variables are expressed as medians with interquartile ranges or depicted as boxplots, with boxes indicating the 25th and 75th percentiles, the median shown as a central line, and whiskers representing the 5th and the 95th percentiles [15]. Normality was assessed using the D’Agostino–Pearson omnibus test [16], and between-group comparisons were performed with the Kruskal–Wallis test [17]. Statistical significance was defined as a two-tailed *p* value < 0.05. Analyses were conducted with GraphPad Prism (version 11.0.2; GraphPad Software, San Diego, CA, USA).

## 3. Results

### 3.1. Search Results

**Figure 1:**
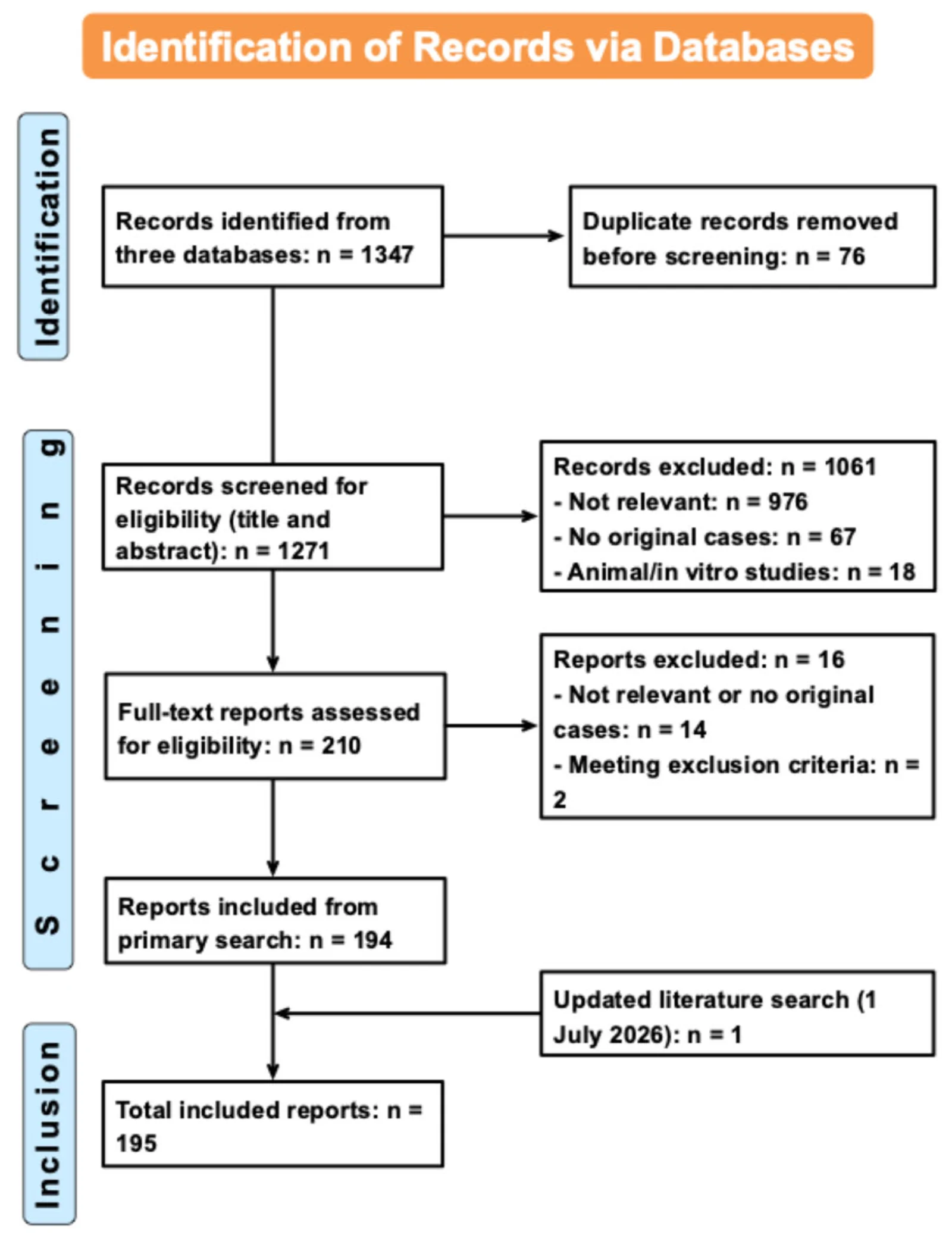
McKittrick–Wheelock syndrome. Flowchart of the literature search.

The study flowchart is shown in Figure 1.

After duplicate reports had been excluded, 195 reports published between 1955 and 2026 and describing 231 patients with McKittrick–Wheelock syndrome were included in the final analysis [18,19,20,21,22,23,24,25,26,27,28,29,30,31,32,33,34,35,36,37,38,39,40,41,42,43,44,45,46,47,48,49,50,51,52,53,54,55,56,57,58,59,60,61,62,63,64,65,66,67,68,69,70,71,72,73,74,75,76,77,78,79,80,81,82,83,84,85,86,87,88,89,90,91,92,93,94,95,96,97,98,99,100,101,102,103,104,105,106,107,108,109,110,111,112,113,114,115,116,117,118,119,120,121,122,123,124,125,126,127,128,129,130,131,132,133,134,135,136,137,138,139,140,141,142,143,144,145,146,147,148,149,150,151,152,153,154,155,156,157,158,159,160,161,162,163,164,165,166,167,168,169,170,171,172,173,174,175,176,177,178,179,180,181,182,183,184,185,186,187,188,189,190,191,192,193,194,195,196,197,198,199,200,201,202,203,204,205,206,207,208,209,210,211,212]. These reports were written in English (n = 150), Spanish (n = 25), German (n = 7), French (n = 2), Italian (n = 2), Turkish (n = 2), Japanese (n = 1), Slovak (n = 1), Hungarian (n = 1), Russian (n = 1), Norwegian (n = 1), Portuguese (n = 1), and Lithuanian (n = 1). Geographically, 76 reports originated from the Americas (United States, n = 55; Mexico, n = 5; Canada, n = 4; Argentina, n = 3; Brazil, n = 2; Chile, n = 2; Colombia, n = 2; Bolivia, n = 1; Puerto Rico, n = 1; Uruguay, n = 1), 33 from Asia (Japan, n = 12; Turkey, n = 6; Taiwan, n = 4; India, n = 3; China, n = 1; Israel, n = 1; Indonesia, n = 1; Iraq, n = 1; Korea, n = 1; Malaysia, n = 1; Philippines, n = 1; Thailand, n = 1), 78 from Europe (United Kingdom, n = 17; Spain, n = 16; Italy, n = 10; Germany, n = 8; Romania, n = 5; France, n = 2; Belgium, n = 2; Denmark, n = 2; Lithuania, n = 2; the Netherlands, n = 2; Austria, n = 1; Bosnia and Herzegovina, n = 1; Bulgaria, n = 1; Croatia, n = 1; Hungary, n = 1; Latvia, n = 1; Norway, n = 1; Poland, n = 1; Portugal, n = 1; Russia, n = 1; Slovakia, n = 1; Sweden, n = 1), one from Africa (Egypt), and seven from Oceania (Australia, n = 6; New Zealand, n = 1). Reporting completeness was classified as excellent in 103 (45%) cases, good in 96 (42%), and acceptable in the remaining 32 (14%) cases.

### 3.2. Findings, Management, Course

Of the 231 patients with McKittrick–Wheelock syndrome, three-quarters were aged 59 years or older, with a slight female predominance, and symptom duration generally exceeded 12 months (Table 2).

Beyond diarrhea (an inclusion criterion), the most common manifestations were fatigue and orthostatic intolerance, whereas weight loss, nausea or vomiting, and hematochezia were less frequent. Tumors were predominantly located within 100 mm of the anal verge (68%) and measured at least 51 mm in maximum diameter, with 78% measuring ≥100 mm. Histologically, most lesions were villous or tubulovillous adenomas (69%), with 23% showing intramucosal carcinoma.

**Figure 2:**
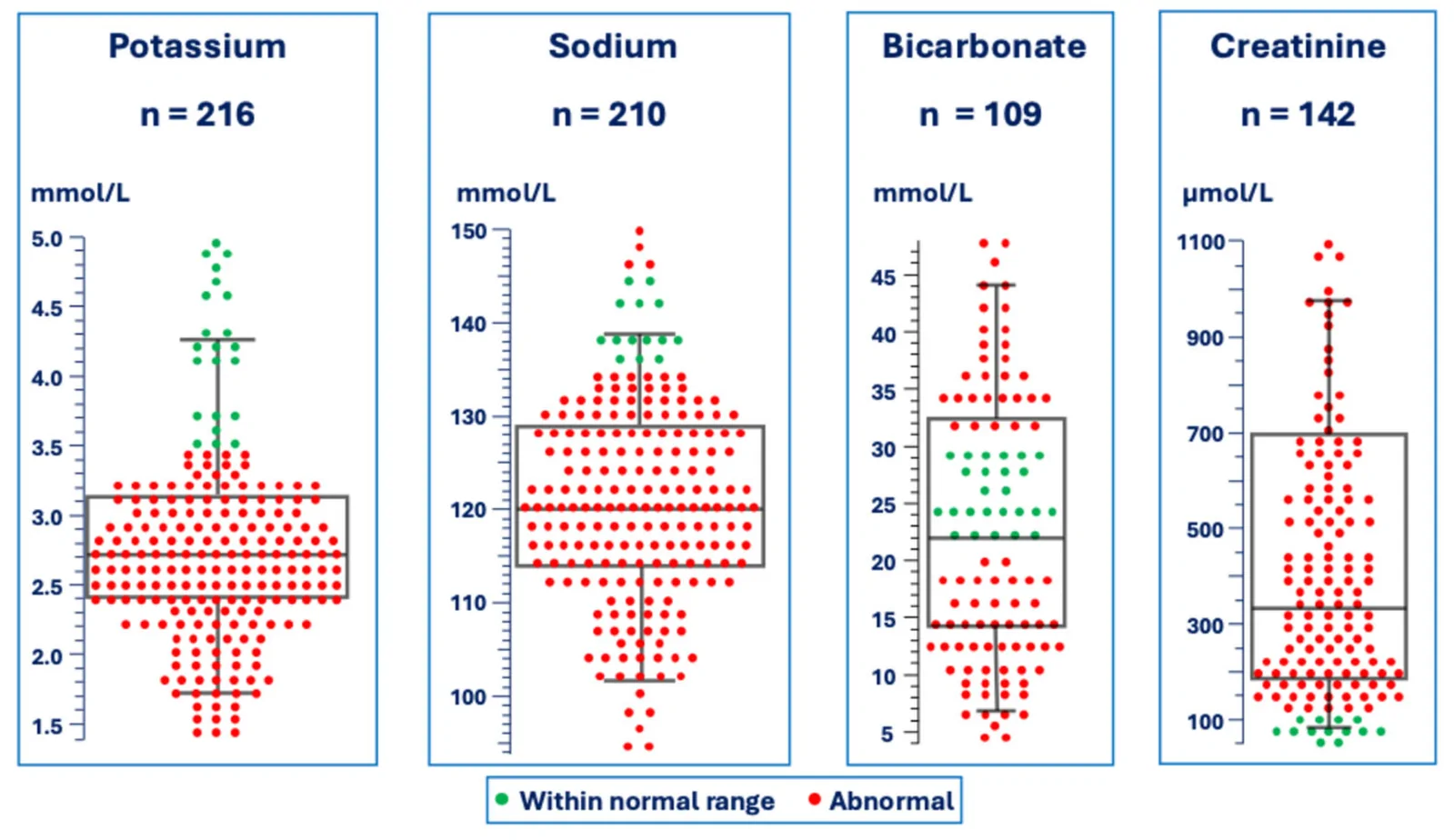
Distribution of potassium (2.7 [2.4–3.1] mmol/L), sodium (120 [114–129] mmol/L), bicarbonate (22 [14–32] mmol/L), and creatinine (338 [184–687] µmol/L) levels in patients with McKittrick–Wheelock syndrome. Data are shown as individual values and boxplots. The lower and upper box boundaries represent the 25th and 75th percentiles, the central line the median, and the whiskers the 5th and 95th percentiles. Green symbols indicate normal values and red symbols abnormal values.

The distribution of electrolyte, acid–base, and kidney parameters is shown in Figure 2.

Potassium levels were available in 216 patients, of whom only 22 (10%) had normal potassium concentrations, while 194 (90%) had hypokalemia. Among the latter, the deficiency was mild in 45 (23%), moderate in 78 (40%), severe in 45 (23%), and profound in 26 (13%) cases. Sodium levels were available in 210 patients: 14 (6.7%) had normal sodium concentrations, whereas 192 (91%) had hyponatremia. Of these, hyponatremia was classified as mild in 34 (18%), moderate in 25 (13%), severe in 43 (22%), and profound in 90 (47%). Mild hypernatremia was identified in 4 (1.9%) cases. Metabolic acid–base status was assessed in 109 patients and was normal in 25 (23%) and abnormal in 84 (77%). Metabolic acidosis, found in 54 (50%) cases, was mild in 14 (26%), moderate in 25 (46%), severe in 13 (24%), and profound in 2 (3.7%). Metabolic alkalosis was identified in 30 (28%) patients and was categorized as mild in 13 (43%), moderate in 8 (27%), severe in 6 (20%), and profound in 3 (10%). Creatinine was ≥101 µmol/L in 129 (91%) cases. Of these, 14 cases (11%) were classified as category 1, 39 (30%) as category 2, 46 (36%) as category 3, and 30 (23%) as category 4. The molar urea-to-creatinine ratio was calculated in 26 individuals with blood urea ≥10 mmol/L and blood creatinine ≤250 µmol/L. The ratio ranged from 51 to 80 in 4 patients (15%) and from 108 to 513 in the remaining 22 (85%).

Compared with patients with metabolic alkalosis, those with metabolic acidosis had substantially lower sodium concentrations (118 [107–123] versus 123 [116–131] mmol/L; *p* = 0.0189) and higher potassium (2.7 [2.4–3.1] versus 2.4 [2.0–2.7] mmol/L; *p* = 0.0051) and creatinine concentrations (619 [415–865] versus 252 [166–534] µmol/L; *p* = 0.0030).

Clinical and laboratory characteristics did not differ significantly between cases with tumor sizes of 51–149 mm and ≥150 mm (Table 3).

A similar figure was observed in cases published during the periods 1955–1999 and 2000–2026 (Table 4).

### 3.3. Mechanisms Underlying Diarrhea

The stool osmotic gap, estimated from stool water sodium and potassium concentrations in eight cases, was uniformly <50 mOsm/kg (range, 4–32; median, 20 mOsm/kg). In seven cases, non-steroidal anti-inflammatory drug therapy was followed by a reported reduction in stool losses.

## 4. Discussion

This patient-level systematic review provides the most comprehensive synthesis of McKittrick–Wheelock syndrome to date and describes a recognizable syndrome of chronic diarrhea, associated with substantial electrolyte and acid–base disturbances, and elevated creatinine concentrations. Patients were usually older adults, symptoms often persisted for more than one year before diagnosis, and tumors were typically large distal colorectal adenomas, most commonly villous or tubulovillous lesions. Another clinically important observation is the long diagnostic delay. Median symptom duration exceeded 12 months, indicating prolonged morbidity before recognition. Chronic diarrhea associated with weakness or orthostatic intolerance should prompt evaluation.

Almost all tumors were located within 29 cm of the anal verge and more than two-thirds within 10 cm. These findings support the clinical value of anorectal examination and timely endoscopic evaluation. No tumor measuring ≤50 mm was reported among patients with a recorded diameter. This observation may reflect selection and reporting bias, and a biological size threshold cannot be inferred. We did not detect differences in the reported biochemical variables between the two size groups, but 78 of 231 patients had no recorded tumor diameter. Overall, the findings cannot establish a plateau in secretory capacity.

Biochemical abnormalities were prominent. Hypokalemia, hyponatremia, and creatinine ≥101 µmol/L were each reported in about 90% of evaluated patients. Disturbances were often marked: 37% of hypokalemic patients had potassium levels ≤2.4 mmol/L, nearly half of hyponatremic patients had sodium levels ≤119 mmol/L, and most patients with creatinine concentrations ≥101 µmol/L had values ≥301 µmol/L.

A further contribution of this review is the delineation of acid–base patterns. Acid–base disorders were present in 77% of assessed cases. Although diarrhea classically causes metabolic acidosis, more than a quarter of assessed patients had metabolic alkalosis [2,213,214]. Both acidosis (bicarbonate was ≤9 mmol/L in 28% of these patients) and alkalosis (bicarbonate ≥40 mmol/L in 30% of these patients) were mostly marked. Metabolic acidosis was associated with lower sodium and higher potassium and creatinine concentrations than metabolic alkalosis.

The findings of our systematic review do not allow us to establish the pathophysiology of kidney dysfunction in McKittrick–Wheelock syndrome. However, the history of chronic diarrhea and the elevated urea-to-creatinine ratio suggest that kidney dysfunction may have been initially triggered by volume depletion.

The literature suggests approaches to correcting volume and electrolyte disturbances and assessing patients for endoscopic, transanal, or surgical tumor removal according to lesion features. This review did not compare treatments or establish the benefit of a specific approach. Nonetheless, available data also suggest that stool osmotic gap was low, and treatment with nonsteroidal anti-inflammatory drugs was followed by a reduction in stool losses. Although based on a very limited number of cases, these findings suggest secretory diarrhea mediated, at least in part, by prostaglandins [12,213]. However, the use of nonsteroidal anti-inflammatory drugs should be cautiously considered given the frequent coexistence of impaired kidney function [215].

This review has several limitations. The source evidence consists almost entirely of case reports and small series; selective publication and selective reporting favor severe or unusual presentations. The reporting-completeness score is not a formal risk-of-bias assessment, and no validated study-level appraisal was performed. Missing baseline creatinine, urine-output data, serial kidney measurements, and follow-up precluded classification of acute kidney injury, chronic kidney disease, recovery, or irreversible damage. Reports span 1955–2026, when laboratory reference ranges, endoscopic practice, care, and reporting standards changed. Thus, the absence of detected differences across the arbitrary year-2000 split cannot establish a stable phenotype over time. The findings of this review describe a recognizable clinical presentation but neither validate diagnostic criteria nor support estimates of risk or prognosis. Similarly, whether earlier recognition improves outcomes could not be evaluated in this review. Finally, agent, dose, and treatment duration of anti-inflammatory drugs were not systematically captured and require future studies to assess their effectiveness and safety.

## 5. Conclusions

McKittrick–Wheelock syndrome is a recognizable, potentially treatable presentation of chronic diarrhea with major electrolyte and acid–base disturbances and frequently elevated creatinine. The case literature supports prompt correction of volume and metabolic abnormalities and evaluation of the underlying colorectal lesion. The available reports cannot establish the mechanism of diarrhea in most patients, classify kidney injury, or determine long-term outcomes.

## Figures and Tables

**Table 1:** McKittrick–Wheelock syndrome. Severity classifications used for biochemical abnormalities and creatinine concentrations in this patient-level systematic review [8,9,10,11,12]. Only the definitions useful for classifying the cases included in the review were used.

**• Hypokalemia (<3.5 mmol/L):** mild: 3.0–3.4; moderate: 2.5–2.9; severe: 2.0–2.4; profound: ≤1.9
**• Sodium disorders (mmol/L)**
** - Hyponatremia** (<135): mild: 130–134; moderate: 125–129; severe: 120–124; profound: ≤119
- **Hypernatremia** (>145): mild: 146–150; moderate: 151–155; severe: 156–160; profound: ≥161
**• Metabolic acidosis (bicarbonate <21 mmol/L; pH <7.36):** mild: 15–20; moderate: 10–14; severe: 5–9; profound: ≤4
**• Metabolic alkalosis (bicarbonate >29 mmol/L; pH >7.44):** mild: 30–34; moderate: 35–39; severe: 40–44; profound: ≥45
**• Descriptive hypercreatininemia categories (µmol/L) ^†^:** category 1: 101–150; category 2: 151–300; category 3: 301–600; category 4: ≥601
**• Possible indicator of volume depletion ^‡^:** molar urea-to-creatinine ratio ≥100, evaluated only when blood urea ≥10 mmol/L and creatinine ≤250 µmol/L.
**• Classification of chronic diarrhea (mOsm/kg)**
- Stool osmotic gap = 290 − 2 × (stool Na^+^ + stool K^+^) *
- Classification: secretory <50; osmotic >125; unclassified 50–125

^†^ Author-defined hypercreatininemia categories. These are not validated grades and do not establish acute kidney injury or chronic kidney disease; ^‡^ A ratio alone does not diagnose prerenal acute kidney injury; * Stool Na^+^ and K^+^ expressed in mmol/L. The name of the categories are in bold.

**Table 2:** Baseline characteristics, clinical presentation, and tumor features of 231 patients with McKittrick–Wheelock syndrome (age range 25–89 years). Data are presented as frequency (percentage) or median [interquartile range]. Percentages were calculated using the number of cases with available data for each variable. * Diarrhea was an inclusion criterion; 231 (100%) is therefore defined by case selection. The name of the main categories are in bold.

**Demographics**	
Age, years	68 [59–74]
Females: males	123: 108
**Presentation**	
Duration of symptoms, months	13 [3–36]
Symptoms	
Diarrhea	231 * (100)
Fatigue	120 (52)
Orthostatic intolerance	115 (50)
Nausea and vomiting	77 (33)
Weight loss	72 (31)
Hematochezia	49 (21)
Abdominal pain	44 (19)
Tumor prolapse	17 (7.4)
**Tumor characteristics**	
Distance from the anal verge; cm	
≤5	66 (30)
6–10	82 (38)
11–15	26 (12)
16–29	36 (17)
≥30	7 (3.2)
Data unavailable	14
Tumor size (mm)	
≤50	0 (0)
51–99	34 (22)
100–149	60 (39)
150–199	41 (27)
≥200	18 (12)
Data unavailable	78
**Histopathological classification**	
Villous adenoma	120 (55)
Tubulovillous adenoma	30 (14)
Tubular adenoma	3 (1.4)
Unclassified adenoma	7 (3.2)
Adenoma with intramucosal carcinoma	50 (23)
Adenocarcinoma	8 (3.7)
Data unavailable	13

**Table 3:** Demographic and biochemical characteristics by reported tumor size in 153 (66%) of 231 patients with McKittrick–Wheelock syndrome. Data are presented as frequency (percentage) or median [interquartile range]. Percentages were calculated using the number of cases with available data for each variable. In patients with unknown tumor size, potassium (2.6 [2.3–2.9] mmol/L), sodium (121 [115–129] mmol/L), bicarbonate (24 [17–30] mmol/L), and creatinine (340 [180–689] µmol/L) concentrations did not differ significantly from those in patients with tumor sizes of 51–149 mm or ≥150 mm.

	Tumor Size				
	51–149 mm n = 94		≥150 mm n = 59		*p* Value
	n *		**n** *		
**Demographics**	94		59		
Females: males		48: 46		35: 24	0.407
Age, years		68 [60–75]		67 [59–73]	0.4876
**Laboratory values**					
Potassium	90		56		
Concentration, mmol/L		2.6 [2.3–3.1]		2.7 [2.3–3.0]	0.8899
Hypokalemia		81 (90)		50 (89)	>0.9999
Sodium	85		51		
Concentration, mmol/L		119 [112–127]		122 [115–128]	0.2350
Hyponatremia		77 (91)		46 (90)	>0.9999
Hypernatremia		2 (2.4)		2 (3.9)	0.6305
Bicarbonate	44		28		
Concentration, mmol/L		22 [14–29]		29 [14–34]	0.1597
Metabolic acidosis		19 (43)		11 (39)	0.8094
Metabolic alkalosis		10 (23)		12 (43)	0.1143
Creatinine, µmol/L	57		36		
Concentration, µmol/L		335 [190–690]		360 [175–670]	0.4523
Creatinine ≥101 µmol/L		50 (88)		33 (92)	0.7354

* Number of patients for whom the information was available.

**Table 4:** Exploratory comparison of reported cases published during 1955–1999 and 2000–2026. Data are presented as frequency (percentage) or median [interquartile range]. The calendar-year-2000 cutoff was chosen pragmatically for an exploratory comparison; non-significant tests do not demonstrate equivalence between periods.

	1955–1999 n = 79		2000–2026 n = 152		
	n *		n *		
**Demographics**	79		152		
Females: males		41 (52): 38 (48)		82 (54): 70 (46)	0.7826
Age, years		65 [59–72]		69 [60–75]	0.0887
**Laboratory values**					
Potassium, mmol/L	71	2.6 [2.2–3.1]	145	2.7 [2.4–3.0]	0.2836
Sodium, mmol/L	68	119 [112–129]	142	120 [113–129]	0.7480
Bicarbonate, mmol/L	33	24 [14–33]	76	18 [11–32]	0.1145
Creatinine, µmol/L	41	414 [256–784]	101	338 [183–644]	0.2148
**Tumor size (mm)**	51		102		0.4357
51–99		11		23	
100–149		18		42	
150–199		14		27	
≥200		8		10	
**Completeness of reporting**	79		152		0.1667
Acceptable		14		18	
Good		34		62	
Excellent		31		72	

* Number of patients for whom the information was available.

## Data Availability

Data sharing is not applicable to this report, as no new data were generated during the study.

## Supplementary Materials

The following supporting information can be downloaded at: https://www.mdpi.com/article/10.3390/jcm15218374/s1, Table S1: PRISMA 2020 checklist.
